# Longitudinal changes in qualitative aspects of semantic fluency in presymptomatic and prodromal genetic frontotemporal dementia

**DOI:** 10.1007/s00415-023-11845-5

**Published:** 2023-07-18

**Authors:** Lize C. Jiskoot, Esther van den Berg, Sascha A. A. M. Laenen, Jackie M. Poos, Lucia A. A. Giannini, Djaina D. Satoer, Judy van Hemmen, Yolande A. L. Pijnenburg, Jet M. J. Vonk, Harro Seelaar

**Affiliations:** 1 Department of Neurology and Alzheimer Center Erasmus MC, Erasmus MC University Medical Center, Room NF-331, Post Box 2040, 3000 CA Rotterdam, The Netherlands; 2 Dementia Research Centre, University College London, London, UK; 3 Department of Neurosurgery, Erasmus University Medical Center, Rotterdam, The Netherlands; 4 Alzheimer Center Amsterdam, Amsterdam University Medical Center, Amsterdam, The Netherlands; 5 Memory and Aging Center, Department of Neurology, University of California, San Francisco, CA USA; 6 Department of Epidemiology, Utrecht University Medical Centre, Utrecht, The Netherlands

**Keywords:** Frontotemporal dementia, Semantic fluency, Neuropsychology, Phenoconversion

## Abstract

**Background:**

The semantic fluency test is one of the most widely used neuropsychological tests in dementia diagnosis. Research utilizing the qualitative, psycholinguistic information embedded in its output is currently underexplored in presymptomatic and prodromal genetic FTD.

**Methods:**

Presymptomatic *MAPT* (*n* = 20) and *GRN* (*n* = 43) mutation carriers, and controls (*n* = 55) underwent up to 6 years of neuropsychological assessment, including the semantic fluency test. Ten mutation carriers became symptomatic (*phenoconverters*). Total score and five qualitative fluency measures (lexical frequency, age of acquisition, number of clusters, cluster size, number of switches) were calculated. We used multilevel linear regression modeling to investigate longitudinal decline. We assessed the co-correlation of the qualitative measures at each time point with principal component analysis. We explored associations with cognitive decline and grey matter atrophy using partial correlations, and investigated classification abilities using binary logistic regression.

**Results:**

The interrater reliability of the qualitative measures was good (ICC = 0.75–0.90). There was strong co-correlation between lexical frequency and age of acquisition, and between clustering and switching. At least 4 years pre-phenoconversion, *GRN* phenoconverters had fewer but larger clusters (*p* < 0.001), and fewer switches (*p* = 0.004), correlating with lower executive function (*r* = 0.87–0.98). Fewer switches was predictive of phenoconversion, correctly classifying 90.3%. Starting at least 4 years pre-phenoconversion, *MAPT* phenoconverters demonstrated an increase in lexical frequency (*p* = 0.009) and a decline in age of acquisition (*p* = 0.034), correlating with lower semantic processing (*r* = 0.90). Smaller cluster size was predictive of phenoconversion, correctly classifying 89.3%. Increase in lexical frequency and decline in age of acquisition were associated with grey matter volume loss of predominantly temporal areas, while decline in the number of clusters, cluster size, and switches correlated with grey matter volume loss of predominantly frontal areas.

**Conclusions:**

Qualitative aspects of semantic fluency could give insight into the underlying mechanisms as to why the “traditional” total score declines in the different FTD mutations. However, the qualitative measures currently demonstrate more fluctuation than the total score, the measure that seems to most reliably deteriorate with time. Replication in a larger sample of FTD phenoconverters is warranted to identify if qualitative measures could be sensitive cognitive biomarkers to identify and track mutation carriers converting to the symptomatic stage of FTD.

## Introduction

Frontotemporal dementia (FTD) is a clinically and pathologically heterogeneous type of early-onset dementia, typically characterized by atrophy of the frontal and/or temporal lobes [1]. The clinical profile of FTD shows behavioural and language disturbances, with cognitive deficits in executive function and relative sparing of memory and visuospatial abilities [2]. Up to 40% of FTD cases have an autosomal dominant pattern of inheritance. Mutations in the progranulin (*GRN*), microtubule-associated protein tau (*MAPT*) and chromosome 9 open reading frame 72 (*C9orf72*) genes are the most common causes [3]. Early diagnosis—albeit difficult due to the heterogeneous symptoms and overlap with other forms of dementia and psychiatric disorders—is essential for proper patient management and planning, non-pharmacological treatment, and patient stratification in upcoming disease-modifying clinical trials [4].

Research in the genetic FTD field has been increasingly moving towards the presymptomatic and early prodromal stages, as the critical time-window for treatment most likely lies prior to overt symptom onset. With promising avenues opening for clinical trials, identifying robust biomarkers is of utmost importance [5]. Previous neuropsychological studies showed that subtle cognitive deficits and decline are present in the presymptomatic stage, and gene-specific cognitive profiles can be detected [6–8]. These findings suggest that neuropsychological assessment in the presymptomatic and early prodromal stages can provide sensitive cognitive markers for FTD.

The semantic fluency test is one of the most widely used tests in neuropsychological assessments. In this brief, easy-to-apply test, people have to generate items from a particular semantic category (e.g., animals, foods) in 1 min [9]. The semantic fluency test presents high sensitivity and specificity for dementia diagnosis [9], with impaired performance found in both symptomatic [10] and presymptomatic FTD [7, 8]. Although the total number of items generated is commonly used to quantify test performance, qualitative, psycholinguistic information embedded in the output can also be investigated, including clusters (number of multiword strings^1^), switches (number of transitions between clusters), age of acquisition (AoA; the age at which a word is learned), and lexical frequency (LF; how often a word occurs in daily language) [12, 13]. Previous research demonstrated the prognostic value of qualitative fluency measures in cognitively healthy subjects at-risk for and in conversion from prodromal to overt Alzheimer’s Dementia (AD) [14, 15]. This approach has been underexplored in presymptomatic and/or prodromal genetic FTD, while this psycholinguistic information may be able to detect the subtle development of FTD’s characteristic language symptoms at an early stage.

The aim of this study was therefore to investigate longitudinal changes in five qualitative aspects of semantic fluency (i.e., number of clusters and switches, cluster size, AoA, and LF) in mutation carriers that developed FTD (*phenoconverters*), presymptomatic mutation carriers, and non-carriers from autosomal dominant *GRN*- and *MAPT*-FTD families. We were specifically interested in the inflection point (i.e., *when* in the disease trajectory) at which the qualitative measures start to deviate from normal. Additionally, we explored the co-correlation between the qualitative measures, and their associations with cognitive decline and grey matter (GM) volume loss, and the prognostic value of decline in qualitative measures in predicting symptomatic onset.

## Methods

### Participants

We included longitudinal data of 118 participants from the FTD Risk Cohort of the Erasmus MC University Medical Center (Rotterdam, the Netherlands). This is an ongoing study in which first-degree family members of FTD patients due to a pathogenic mutation are followed on a 1- or 2-year basis [16]. Participants were recruited between February 2010 and October 2019. DNA genotyping at study entry assigned participants to the mutation carrier (*n* = 63; *MAPT* *n* = 20, *GRN* *n* = 43) or non-carrier group (controls; *n* = 55). Upon study entry, all mutation carriers were presymptomatic according to clinical diagnostic criteria for FTD [2, 17, 18], and had global CDR^®^-plus-NACC-FTLD [19] scores of 0. Ten mutation carriers (*MAPT* *n* = 6, *GRN* *n* = 4) developed symptoms during follow-up (phenoconverters). Diagnoses were made in multidisciplinary consensus meetings, using information from the standardized clinical assessment (see below). Phenoconverters met the following criteria: [i] progressive deterioration of behaviour, language and/or motor functioning; [ii] functional decline, evidenced by multiple study visits with global CDR^®^-plus-NACC-FTLD [19] ≥ 0.5 without reversing back to 0; and [iii] cognitive decline, evidenced by ≥ 1.5 SD below age-, sex- and education-specific means in ≥ 1 domain on neuropsychological assessment. Eight phenoconverters had clinical features of bvFTD (*MAPT* *n* = 6, *GRN* *n* = 2), and two had non-fluent variant PPA (*GRN* *n* = 2). The presymptomatic mutation carriers that did not develop FTD symptoms are referred to as *non-converters* (*n* = 53).

### Clinical assessment

Every 1–2 years, all participants underwent a standardized clinical assessment, consisting of a structured interview with the participant and a knowledgeable informant (incorporating the CDR^®^-plus-NACC-FTLD [19]), medical history taking, neurological examination, neuropsychological assessment, and brain MRI. The neuropsychological assessment consisted of cognitive screening tests (Mini-Mental State Examination, MMSE, and Frontal Assessment Battery, FAB), and tests within the major cognitive domains. Neuropsychiatric symptoms were assessed with the Beck’s Depression Inventory (BDI) and the brief questionnaire form of the Neuropsychiatric Inventory (NPI-Q) (see [7, 8] for full test battery).

### Qualitative fluency measures

The semantic fluency task was part of the neuropsychological assessment at each study visit. In this task, participants were asked to verbally generate as many different animals as possible in 60 s [20]. The total score is the number of animals produced minus errors. Additionally, we calculated five qualitative fluency measures from the output: LF, AoA, number of clusters, cluster size and number of switches (Box 1, Appendix 1 for scoring guidelines).

Box 1. The 5 qualitative measures generated from the semantic fluency output

- *Lexical Frequency (LF)*—a measure of how often a particular word occurs in daily language. Higher LF indicates worse performance (i.e. more use of high frequency words). Each generated word was paired with its log-transformed LF value from the SUBTLEX-NL database, so that it reflects the natural logarithmic value of how often a word occurs per one million Dutch words. The SUBTLEX-NL database was based on 44 million words from Dutch film and TV subtitles [21]. For this study, the mean LF per participant per study visit was used for subsequent analysis.
- *Age of Acquisition (AoA)*—reflects the age at which a word is typically learned based on ratings of adults. Lower AoA indicates worse performance (i.e. loss of later acquired words). Each generated word was paired with its AoA from a database containing 30,000 Dutch words [22]. For this study, the mean AoA per participant per study visit was used for subsequent analysis.
- *Number of clusters*—constitutes the number of related multiword strings (e.g., pets, birds), with a minimum of at least two successive words within a semantic subcategory [13]. A lower number of clusters indicates worse performance.
- *Cluster size*—is the sum of all clustered words. The mean cluster size per participant per study visit was used for subsequent analysis (i.e. total cluster size divided by the number of clusters). Lower cluster size indicates worse performance.
- *Number of switches*—defined as the number of transitions between different clusters, between clustered and non-clustered words (i.e. transitions from one associative strategy to none at all), or between non-clustered and other non-clustered words [13]. A lower number of switches indicates worse performance.

### Study design

**Fig. 1:**
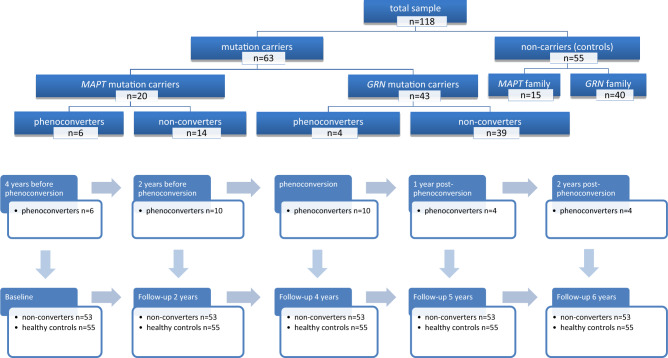
Subject sample and study design. The total sample (*n* = 118) was divided into mutation carriers (*n* = 63) and non-carriers (healthy controls; *n* = 55), the mutation carrier group was split into phenoconverters (*n* = 10) and non-converters (*n* = 53). The original data was restructured for phenoconverters, so that there were five time points: 4 years before phenoconversion, 2 years before phenoconversion, phenoconversion, 1 year post-phenoconversion and 2 years post-phenoconversion. Four years before phenoconversion data was available for only 6 phenoconverters, as the other four phenoconverters developed symptoms between baseline and the first follow-up visit, and therefore no data 4 years prior to phenoconversion were available. One and 2 years after phenoconversion data was available for only 4 phenoconverters; the other 6 were either lost to follow-up, as they were clinically too impaired to undergo neuropsychological testing or passed away (*n* = 4), or converted recently so that follow-up data post-phenoconversion was not available at this time point (*n* = 2). The data of phenoconverters was compared to respectively baseline, and follow-up after 2, 4, 5 and 6 years in non-converters and healthy controls. The remaining six converters were either lost to follow-up, as they were clinically too impaired to undergo neuropsychological testing or passed away (*n* = 4), or converted recently so that follow-up data post-phenoconversion was not available at this time point (*n* = 2). The data were compared to the 5-year follow-up data of non-converters and controls

Phenoconverters, non-converters and controls were compared at five time points: baseline, and follow-up after 2, 4, 5 and 6 years, for the purpose of this study restructured as (Fig. 1):

1. *Four years before phenoconversion*—data were available for six phenoconverters. The other four developed symptoms between baseline and the first follow-up visit, therefore no data 4 years prior to phenoconversion were available. The data were compared to the baseline data of non-converters and controls.
2. *Two years before phenoconversion*—data were available for all ten phenoconverters. The data were compared to the 2-year follow-up data of non-converters and controls.
3. *Phenoconversion*—data were available for all ten phenoconverters. The data were compared to the 4-year follow-up data of non-converters and controls.
4. *One year after phenoconversion*—data were available for four phenoconverters. The other six were clinically too impaired to undergo neuropsychological testing or passed away (*n* = 4), or converted recently, therefore no data 1 year after phenoconversion were available yet (*n* = 2). The data were compared to the 5-year follow-up data of non-converters and controls.
5. *Two years after phenoconversion—*data were available for four phenoconverters. The other six were clinically too impaired to undergo neuropsychological testing or passed away (*n* = 5), or converted recently therefore no data 1 year after phenoconversion were available yet (*n* = 1). The data were compared to the 6-year follow-up data of non-converters and controls.

### MRI acquisition and (pre)processing

On each study visit, we performed volumetric T1-weighted MRI scanning on a Philips 3 T Achieva MRI scanner (Philips Medical Systems, Best, the Netherlands) using either an 8- or 32-channel SENSE head coil and the following scan parameters: inversion/repetition time = 933/2200 ms, flip angle = 8°, voxel size = 1.1 × 1.1 × 1.1 mm, matrix size = 256 × 256 × 208, total scan time = 4.43 min. All scans underwent visual quality control. The DICOM images were subsequently corrected for gradient nonlinearity distortions and converted to NifTI format. They were then pre-processed in the Voxel-Based Morphometry (VBM) pipeline in Statistical Parametric Mapping 12 (SPM12; Functional Imaging Laboratory, University College London, London, UK; www.fil.ion.ucl.ac.uk/spm) implemented in Matlab R2018a (Mathworks, USA). First, the T1-weighted images were normalized to a template space and segmented into GM, white matter (WM) and cerebrospinal fluid (CSF), after which they were rigidly aligned. We calculated total intracranial volume (TIV) by adding GM, WM and CSF. Second, the segmentations were spatially normalized to a DARTEL template by applying the flow fields of all the individual scans. Images were smoothed using a 6 mm full width at half maximum (FWHM) isotropic Gaussian kernel. After every preprocessing step, images were visually inspected.

### Statistical analysis

We performed statistical analyses using SPSS Statistics 25.0 (IBM Corp., Armonk, NY). Alpha was set at 0.05 across all comparisons (two-tailed). We compared continuous demographic data between groups using one-way ANOVAs for normally distributed data (with Bonferroni post-hoc tests), or Kruskal–Wallis tests for non-normally distributed data (with Mann–Whitney *U* post-hoc tests). We analysed between-group differences in sex and gene with Pearson *Χ*^2^ tests. Interrater reliability was explored using intraclass correlation analysis. We assessed the co-correlation of the five qualitative measures at each time point by means of principal component analysis using Varimax rotation. Only factors accounting for 3% or more of variance and Eigenvalues > 1 were retained. Factor loadings were only considered meaningful when *r* > 0.450, and any item that did not load sufficiently onto a factor was removed [23]. For ease of interpretation, we first converted raw fluency and relevant neuropsychological measures (Boston Naming Test (BNT), Semantic Association Test (SAT), Trailmaking test B (TMT-B), Stroop colour-word test card III) into z-scores by subtracting the mean of controls from each individual’s raw score at that time point, divided by the SDs of controls at that time point. We then used multilevel linear regression modeling to investigate longitudinal decline in total and qualitative fluency measures. We performed two separate analyses to assess longitudinal change in qualitative fluency measures per [i] clinical status (phenoconverters, non-converters, controls) and [ii] gene (*MAPT*, *GRN*). We entered [i] or [ii], time, and first-order interactions, with age, sex, education and total number of words generated as covariates. Assumptions were checked (non-linearity, dependence of errors, outliers, heteroscedasticity). Significant interactions between covariates and dependent variables were included in the model. We based the covariance structure (Toeplitz Heterogeneous) on the lowest Akaike Information Criterion (AIC), as a lower AIC indicates a better model fit. We included the random intercept as this model presented with a lower AIC. For converters, we calculated deltas of the standardized values for the [i] qualitative fluency measures and [ii] relevant neuropsychological tests between restructured time-point 1 in the six phenoconverters that had data 4 years before phenoconversion, or time-point 2 in the four phenoconverters that only had data 2 years before phenoconversion, and time-point 3 (phenoconversion). We then explored the association between the delta fluency measures and delta neuropsychological tests (corrected for age, sex and education) using partial correlations. Change over time maps were generated by subtracting the GM maps calculated at time-point 3 (phenoconversion) from the maps calculated at time-point 1 or 2 in SPM12. We then explored the relationship between the delta qualitative fluency measures and delta GM maps by means of multiple regression models. Age, sex, TIV, and head coil were entered as covariates. We set the statistical threshold at *p* < 0.05, adjusted for multiple comparisons with familywise error (FWE) correction. Lastly, to investigate classification abilities of these delta z-scores, we performed binary logistic regression analyses. Assumptions were checked (non-linearity, dependence of errors, outliers, multicollinearity). The models were selected with a forward stepwise method according to the likelihood ratio test and applying the standard *p-*values for variable inclusion (0.05) and exclusion (0.10). Goodness of fit was evaluated with the HL *Χ*^2^ test, with Nagelkerke *R*^2^ as measure of effect size. The analyses were adjusted for age, sex, education, and total number of words generated. All models were corrected for multiple comparisons (Bonferroni).

## Results

### Demographics and clinical data

Demographic and clinical data are shown in Table 1. There were no differences between phenoconverters, non-converters, and controls in age [*F*(2,117) = 0.212, *p* = 0.809], sex [*X*(2) = 1.568, *p* = 0.457], gene [*X*(2) = 4.830, *p* = 0.089] or education level [*F*(2,117) = 1.290, *p* = 0.279]. There were no differences between *MAPT* and *GRN* phenoconverters in age [*F*(2,9) = 2.966, *p* = 0.123], sex [*X*(2) = 0.524, *p* = 0.262] or education level [*F*(2,9) = 0.022, *p* = 0.945]. We found no differences between phenoconverters, non-converters, and controls regarding baseline MMSE [*F*(2,117) = 0.229, *p* = 0.796], FAB [*F*(2,40) = 2.504, *p* = 0.095], BDI [*F*(2,116) = 0.607, *p* = 0.547] or NPI-Q [*F*(2,73) = 0.031, *p* = 0.969]. There were no differences between *MAPT* and *GRN* phenoconverters in these measures (all *p* > 0.05). There were no differences in neuropsychological test scores between group at study entry (all *p* > 0.05).

**Table 1:** Demographic and clinical data per subgroup

	Phenoconverters (*n* = 10)	Non-converters (*n* = 53)	Controls (*n* = 55)
Age at study entry, y	47.9 (9.3)	46.7 (10.6)	48.1 (12.0)
Sex, female (%)	5 (50.0)	33 (62.3)	28 (50.9)
Gene in family	*MAPT* = 6 *GRN* = 4	*MAPT* = 14 *GRN* = 39	*MAPT* = 15 *GRN* = 40
Mutation in family	*P301L* = 3, *G272V* = 3, *S82fs* = 4	*P301L* = 7, *G272V* = 1, *R406W* = 1, *L315R* = 2, *S320F* = 2, *D252Y* = 1, *S82fs* = 25, *Q125X* = 10, *V411fs* = 2, *Asp254fs* = 1, *D254fs* = 1	*P301L* = 7, *G272V* = 3, *R406W* = 2, *L315R* = 1, *N296del* = 2, *S82fs* = 23, *Q125X* = 13, *V411fs* = 1, *Asp254fs* = 1, *D254fs* = 1, *Q249X* = 1
Clinical diagnosis	bvFTD = 8, nfvPPA = 2	N/A	N/A
Education (level)*	5.7 (0.8)	5.3 (1.2)	5.1 (1.0)
Clinical data at study entry			
MMSE [0–30]	29.3 (0.8)	29.3 (1.1)	29.2 (1.2)
FAB [0–18]	15.0 (0)	17.2 (0.9)	16.5 (1.5)
BDI [0–63]	5.6 (11.2)	4.4 (5.1)	3.7 (4.4)
NPI-Q [0–36]	0.5 (0.8)	1.4 (18.9)	0.7 (2.6)
BNT [0–60]	54.3 (5.3)	54.0 (4.9)	53.6 (4.3)
SAT verbal [0–30]	27.7 (1.5)	27.8 (1.7)	27.8 (1.3)
TMT-B, seconds	60.4 (22.6)	62.2 (30.0)	67.9 (33.7)
Stroop card III, seconds	94.1 (27.3)	89.5 (23.1)	95.7 (37.9)

Values indicate mean ± SD or *n* (%)

Abbreviations: *MAPT* microtubule-associated protein tau, *GRN* progranulin, *C9orf72* chromosome 9 open reading frame 72, *bvFTD* behavioural variant frontotemporal dementia, *nfvPPA* non-fluent variant primary progressive aphasia, *N/A* not applicable, *MMSE* Mini-Mental State Examination, *FAB* Frontal Assessment Battery, *BDI* Beck’s Depression Inventory, *NPI-Q* neuropsychiatric questionnaire, *BNT* Boston Naming Test, *SAT* Semantic Association Test, *TMT* Trail Making Test

*Dutch educational system categorized into levels from 1 = less than 6 years of primary education to 7 = academic schooling: Duits A KR. Schatten van het premorbide functioneren. In: Hendriks et al. [24]

### Interrater reliability

Two independent raters (LCJ, SAAML), blinded to participant’s clinical and genetic status, scored the number of clusters, cluster sizes, and number of switches (see Methods—Qualitative fluency measures, and Appendix 1). The interrater reliability of all three measures was considered ‘good’ (i.e., intraclass correlation coefficients 0.75–0.90) [25]: number of clusters 0.81 [95% CI 0.76–0.85], cluster sizes 0.85 [95% CI 0.81–0.87], and number of switches 0.85 [95% CI 0.81–0.88].

### Co-correlation between the qualitative measures

Appendix 2 shows the results of the principal component analysis on the five qualitative measures per time-point. Based on our criteria and visual inspection of the scree plot, we could extract two components. The first component explained between 39.5 and 49.7% of the total variance, and both LF and AoA had high loadings (*r* > 0.900). The second factor explained between 29.0 and 43.3% of the total variance, and both the number of clusters and the number of switches, and at some time-points also the cluster size, had high loadings (*r* > 0.59).

### Longitudinal qualitative fluency trajectories

**Fig. 2:**
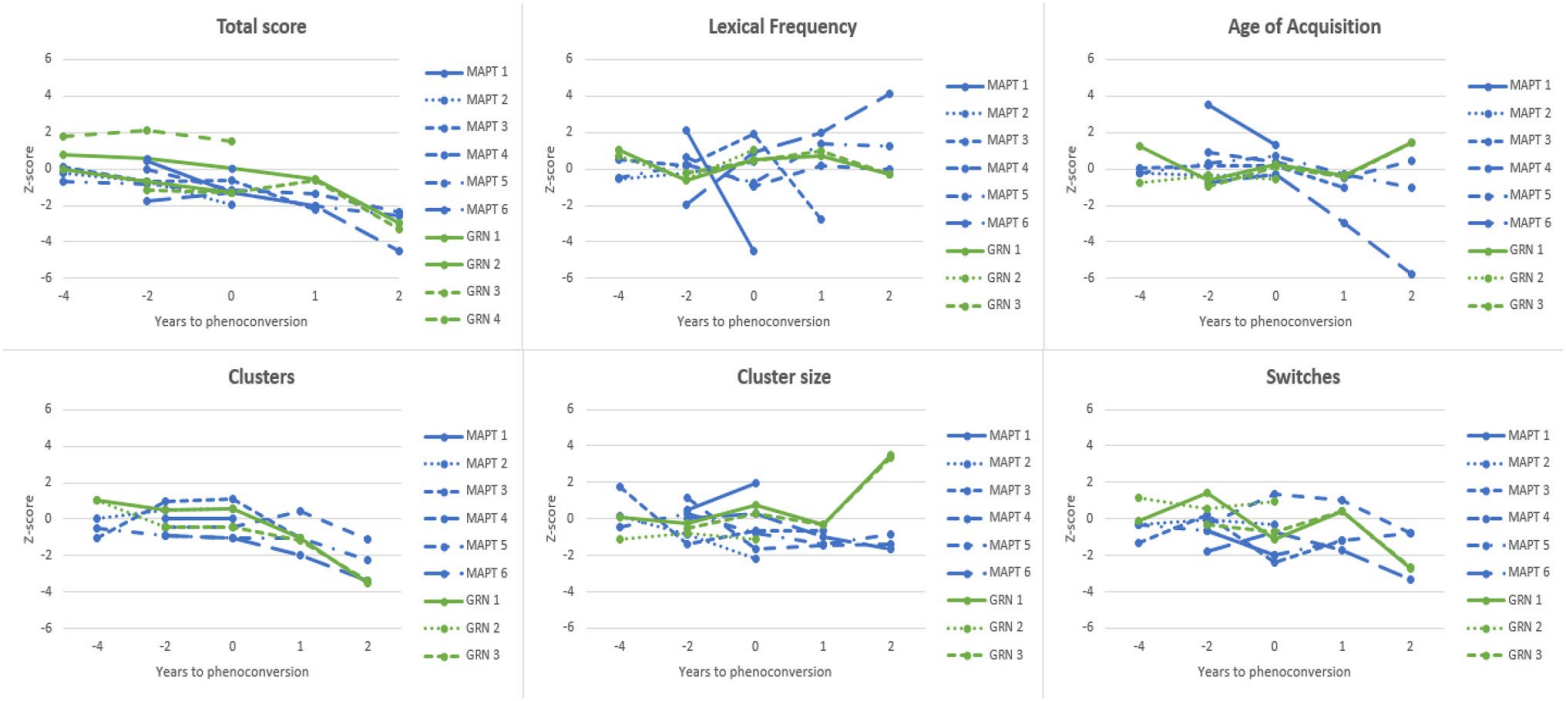
Individual verbal fluency trajectories in the ten FTD phenoconverters. Lines represent the individual longitudinal changes (years to phenoconversion, X-axis) in semantic fluency total scores, lexical, age of acquisition, cluster count and size, and switches (z-scores, Y-axis) in the 10 phenoconverters. The 6 *MAPT* phenoconverters are displayed in blue, the 3 *GRN* phenoconverters in green. Only the total score was available for phenoconverter 10 (*GRN* 4). Abbreviations: *MAPT* microtubule-associated protein tau, *GRN* progranulin

The inflection points and longitudinal trajectories of the fluency measures are shown in Table 2. The individual trajectories in phenoconverters are displayed in Fig. 2. Phenoconverters had declining total scores from at least 4 years pre-phenoconversion (*p* < 0.001), while there was no decline in non-converters or controls (*p* > 0.05). As can be noted from Fig. 2, the qualitative measures demonstrated more noise in their fluctuation than the total score. Phenoconverters had higher LF from 4 years pre-phenoconversion in comparison to controls (*p* = 0.016). Also the number of clusters (*p* < 0.001) and switches (*p* = 0.004) started to decline at this point. AoA declined from phenoconversion onwards (*p* = 0.050). No longitudinal change was found for cluster size (*p* > 0.05). There was no change in qualitative fluency measures in non-converters or controls (*p* > 0.05). Different inflection points and longitudinal trajectories were found for *GRN* and *MAPT* phenoconverters. At least 4 years pre-phenoconversion, *GRN* phenoconverters started producing fewer words in comparison to controls (*p* = 0.005). Moreover, they started producing fewer but larger clusters (both *p* < 0.001), and used fewer switches (*p* = 0.004). *GRN* phenoconverters had larger cluster sizes than *MAPT* phenoconverters (*p* < 0.001). LF and AoA did not change in *GRN* phenoconverters compared to controls (*p* > 0.05). Starting at least 4 years pre-phenoconversion, *MAPT* phenoconverters produced fewer words than controls (*p* < 0.001). Moreover, LF started to increase (*p* = 0.007), while AoA (*p* = 0.034), the number of clusters (*p* = 0.009), and cluster size (*p* = 0.010) declined. The number of switches did not change (*p* > 0.05).

**Table 2:** Longitudinal trajectories of the total score and qualitative fluency measures in phenoconverters and non-converters

Time point	Group	*n*	Total score		LF		AoA		Clusters		Cluster size		Switches	
			Mean	SD	Mean	SD	Mean	SD	Mean	SD	Mean	SD	Mean	SD
4 years before phenoconversion	Phenoconverters (total)	6	**0.29**	**0.87**	**− 0.24**	**0.46**	0.03	0.74	**0.11**	**0.91**	0.08	1.07	**− 0.19**	**0.87**
	*MAPT* Phenoconverters	3	**− 0.27**	**0.42**	**− 0.19**	**0.56**	**− 0.11**	**0.16**	**− 0.50**	**0.51**	**− 0.52**	**0.83**	− 0.66	0.56
	*GRN* Phenoconverters	3	**0.84**	**0.91**	− 0.33	0.42	0.25	1.40	**1.03**	**0.00**	**0.48**	**1.16**	**0.51**	**0.86**
	Non-converters	51	**− **0.20	0.95	0.11	0.99	− 0.23	0.99	− 0.21	0.88	− 0.02	0.80	− 0.21	0.80
2 years before phenoconversion	Phenoconverters (total)	10	**− 0.27**	**1.08**	**− 0.04**	**1.09**	− 0.04	0.77	**− 0.14**	**0.65**	− 0.21	0.77	**− 0.18**	**0.88**
	*MAPT* phenoconverters	6	**− 0.59**	**0.73**	**0.17**	**1.32**	**0.26**	**0.77**	**− 0.14**	**0.75**	**− 0.05**	**0.92**	− 0.53	0.70
	*GRN* phenoconverters	4	**0.22**	**1.44**	− 0.47	0.21	− 0.63	0.29	**− 0.14**	**0.53**	**− 0.52**	**0.26**	**0.53**	**0.87**
	Non-converters	42	**− **0.12	0.74	− 0.03	1.02	0.06	1.04	0.04	1.03	− 0.26	0.86	0.17	1.11
Phenoconversion	Phenoconverters (total)	10	**− 0.86**	**0.99**	**− 0.12**	**1.85**	**0.31**	**1.32**	**− 0.12**	**0.75**	− 0.35	1.29	**− 0.77**	**1.28**
	*MAPT* phenoconverters	6	**− 1.27**	**0.43**	**− 0.50**	**2.22**	**0.49**	**1.61**	**− 0.12**	**0.87**	**− 0.51**	**1.48**	− 1.00	1.39
	*GRN* phenoconverters	4	**− 0.25**	**1.35**	0.65	0.33	− 0.05	0.43	**− 0.12**	**0.61**	**− 0.01**	**0.97**	**− 0.31**	**1.09**
	Non-converters	38	0.20	0.94	0.08	0.86	− 0.04	0.90	0.07	1.20	0.26	1.17	0.03	1.39
1 year after phenoconversion	Phenoconverters (total)	5	**− 1.53**	**0.76**	**− 0.13**	**1.83**	**− 0.02**	**1.24**	**− 0.70**	**0.74**	− 0.97	0.57	**− 0.22**	**1.12**
	*MAPT* phenoconverters	3	**− 1.86**	**0.44**	**− 0.43**	**2.13**	**0.09**	**1.49**	**− 0.58**	**0.85**	**− 1.19**	**0.46**	− 0.43	1.27
	*GRN* phenoconverters	2	**− 0.59**	**0.08**	0.85	0.15	− 0.37	0.04	**− 1.11**	**0.06**	**− 0.33**	**0.02**	**0.42**	**0.02**
	Non-converters	13	0.55	1.52	− 0.12	1.08	0.00	1.21	0.08	1.03	0.47	1.56	0.07	1.02
2 years after phenoconversion	Phenoconverters (total)	5	**− 3.15**	**0.86**	**0.94**	**1.87**	**− 0.54**	**3.03**	**− 2.72**	**1.03**	0.58	2.61	**− 2.07**	**1.19**
	*MAPT* phenoconverters	3	**− 3.13**	**1.12**	**1.77**	**2.10**	**− 1.87**	**3.43**	**− 2.24**	**1.13**	**− 1.31**	**0.43**	− 1.63	1.45
	*GRN* phenoconverters	2	**− 3.17**	**0.23**	− 0.30	0.02	1.45	0.03	**− 3.44**	**0.09**	**− 3.42**	**0.12**	**− 2.72**	**0.05**
	Non-converters	28	0.26	1.33	− 0.27	1.25	0.53	1.36	0.44	0.99	− 0.16	0.64	0.24	0.82

Values indicate: z-scores (i.e. individual test score minus the mean of the healthy controls, divided by the standard deviation of the healthy controls per time point). Data from healthy controls are omitted from this table as their mean z-score is zero and their standard deviation is one by default. Significant values (*p* < 0.05) are expressed in bold

Abbreviations: *SD* standard deviation, *LF* lexical frequency, *AoA* age of acquisition, *MAPT* microtubule-associated protein tau, *GRN* progranulin

### Associations with cognitive decline

Partial correlation coefficients between the fluency measures and relevant neuropsychological tests are shown in Table 3. In phenoconverters, decline on the SAT verbal correlated with an increase in LF (*p* = 0.031) and a decline in AoA (*p* = 0.037). Worse performance on TMT-B (*p* = 0.031) and Stroop-III (*p* = 0.026) correlated with an increase in cluster size, while worse performance on Stroop-III correlated with a decline in the number of switches (*p* = 0.031). In *GRN* phenoconverters, decline on the SAT verbal correlated with a decline in AoA (*p* = 0.020). Worse performance on Stroop-III correlated with a decline in the number of clusters (*p* = 0.022), and an increase in cluster size (*p* = 0.014), while decline on TMT-B correlated with a decline in the number of switches (*p* = 0.024). In *MAPT* phenoconverters, decline on the SAT verbal correlated with an increase in LF (*p* = 0.015) and a decline in AoA (*p* = 0.014).

**Table 3:** Partial correlations between decline in fluency measures and relevant cognitive tests

Group	Measure	BNT	SAT verbal	TMT-B	Stroop card III
Phenoconverters total group (*n* = 10)	LF	− 0.10	**− 0.71**	0.03	− 0.20
	AoA	0.17	**0.70**	− 0.30	− 0.10
	Clusters	0.03	0.44	0.27	0.59
	Cluster size	0.36	0.25	**− 0.71**	**− 0.73**
	Switches	0.16	0.38	0.38	**0.72**
*GRN* phenoconverters (*n* = 4)	LF	0.52	0.96	0.61	0.94
	AoA	− 0.72	**− 0.99**	− 0.79	− 0.83
	Clusters	− 0.10	0.60	0.02	**0.88**
	Cluster size	− 0.41	− 0.92	− 0.51	**− 0.98**
	Switches	0.55	− 0.97	**0.87**	0.93
*MAPT* phenoconverters (*n* = 6)	LF	− 0.21	**− 0.90**	0.44	− 0.51
	AoA	0.22	**0.90**	− 0.12	0.66
	Clusters	0.34	0.77	− 0.37	0.32
	Cluster size	0.30	0.26	− 0.68	− 0.07
	Switches	0.15	0.46	0.65	0.59

Values indicate: partial correlation coefficients (corrected for age, sex and education level). Significant correlations (*p* < 0.05) are expressed in bold

Abbreviations: *BNT* Boston Naming Test, *SAT* Semantic Association Test, *TMT* Trail Making Test, *MAPT* microtubule-associated protein tau, *GRN* progranulin

### Associations with GM volume loss

**Fig. 3:**
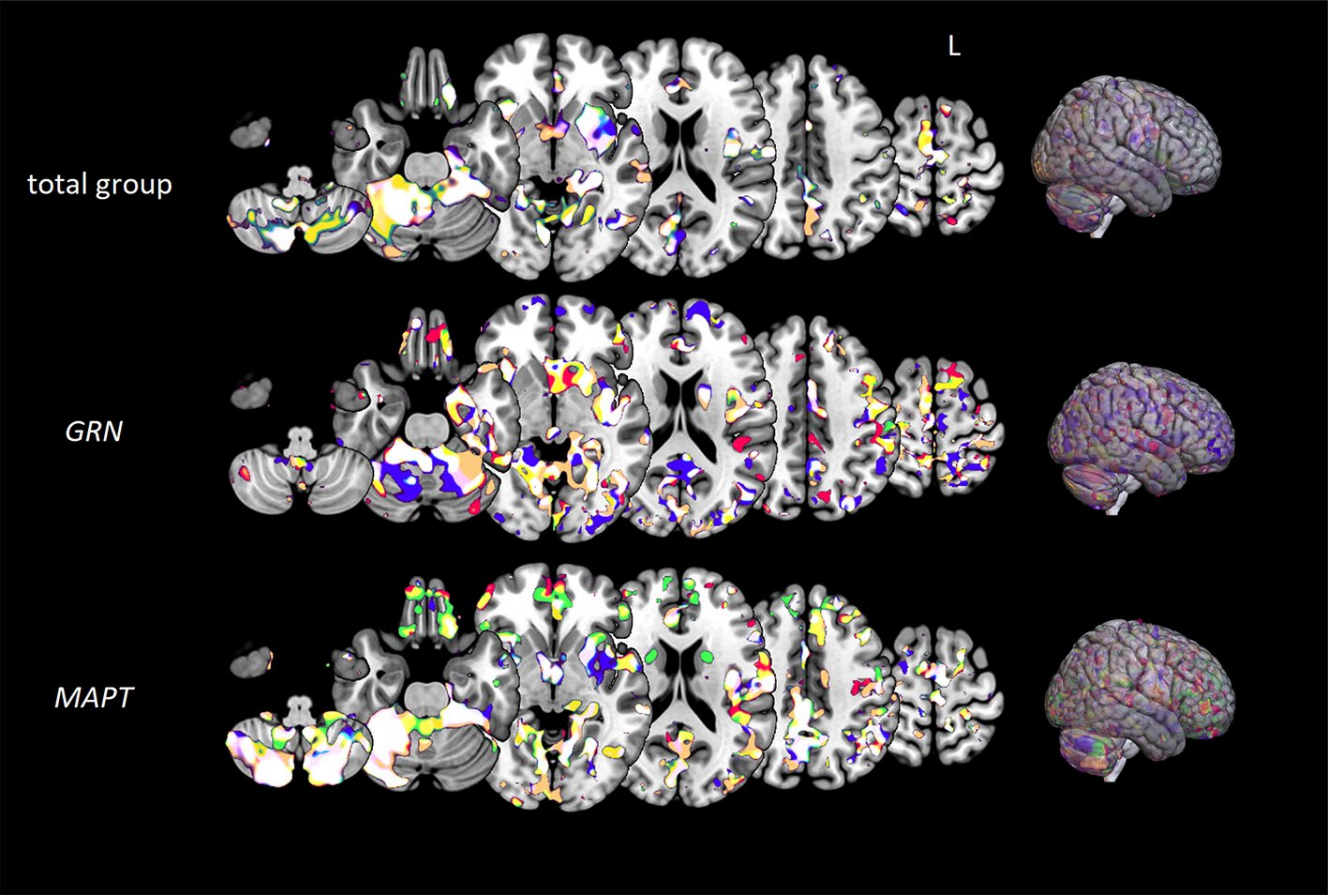
Grey matter atrophy patterns associated with lower qualitative fluency performance. VBM analyses demonstrated grey matter volume loss to be associated with lower performance in lexical frequency (red), age of acquisition (green), clusters (blue), cluster size (yellow), and switches (copper) in the total group of phenoconverters (top), *GRN* phenoconverters (middle), and *MAPT* phenoconverters (bottom). We set the statistical threshold at *p* < 0.05 (FWE-corrected). Abbreviations: *L* left, *GRN* progranulin, *MAPT* microtubule-associated protein tau

The relationships between the qualitative fluency measures and GM volume loss are displayed in Fig. 3 and Appendix 3. In the total group of phenoconverters, worse performance on all five qualitative fluency measures was associated with GM volume loss in large overlapping areas spanning the frontal (e.g., middle frontal gyrus) and temporal lobes (e.g., middle and superior temporal gyrus), and the anterior insular and cingulate cortices (*p*_FWE-corrected_ < 0.05). In *GRN* phenoconverters, an increase in LF and a decline in AoA were associated with GM volume loss of the cerebellum and predominantly temporal areas (e.g., medial temporal lobe, inferior temporal gyrus). A decline in the number of clusters, cluster size, and the number of switches correlated with GM volume loss of the cerebellum, insular cortex, and putamen (*p*_FWE-corrected_ < 0.05). In *MAPT* phenoconverters, an increase in LF and a decline in AoA were associated with GM volume loss of the cerebellum and predominantly temporal areas (e.g., anterior temporal pole, inferior temporal gyrus). A decline in the number of clusters, cluster size, and the number of switches correlated with GM volume loss of the cerebellum and predominantly frontal areas (e.g., middle and superior frontal gyrus, frontal pole) (*p*_FWE-corrected_ < 0.05).

### Classification abilities of qualitative fluency measures

Decline in the total score differentiated between phenoconverters and non-converters [*X*^2^(1) = 7.669, *p* = 0.006] and controls [*X*^2^(1) = 7.643, *p* = 0.006]. This was primarily driven by *MAPT* phenoconverters, as it differentiated well between this group and non-converters [*X*^2^(1) = 5.919, *p* = 0.015] and controls [*X*^2^(1) = 5.902, *p* = 0.015], but not between *GRN* phenoconverters, non-converters, and controls (*p* > 0.05). A decline in switches was predictive of phenoconversion in *GRN* [*X*^2^(1) = 5.069, *p* = 0.024], correctly classifying 90.3% of cases; a decline in cluster size was predictive of phenoconversion in *MAPT* [*X*^2^(1) = 3.894, *p* = 0.048], correctly classifying 89.3% of cases.

## Discussion

This study examined longitudinal changes in qualitative aspects of the semantic fluency task in a large cohort of FTD phenoconverters, presymptomatic mutation carriers, and non-carriers from *GRN*- and *MAPT*-FTD families. Phenoconverters showed a decline in the total score from at least 4 years pre-phenoconversion, with individually-varying inflection points and longitudinal trajectories in qualitative fluency measures in *GRN* and *MAPT*. At least 4 years pre-phenoconversion, *GRN* phenoconverters started producing fewer but larger clusters, and switched less between clusters, which was correlated with executive dysfunction. A decline in switching was predictive of phenoconversion. At least 4 years pre-phenoconversion, *MAPT* phenoconverters demonstrated an increase in LF and a decline in AoA, which was correlated with semantic deficits. A decline in cluster size was predictive of phenoconversion. Increase in LF and decline in AoA were associated with GM volume loss of predominantly temporal areas, while decline in the number of clusters, cluster size, and switches correlated with GM volume loss of predominantly frontal areas.

The semantic fluency total score is strongly intertwined with the qualitative aspects of the task. For accurate and timely word retrieval, both AoA and LF [26], and clustering and switching components [27] are required. Coinciding with the results of our principal component analysis, in which we found a AoA-LF component and a clusters-switches component, previous studies found strong correlations between AoA and LF, and between the number of clusters and the number of switches. With respect to the relation between AoA and LF, correlations have found to be high in natural languages, as early-acquired words tend to occur more frequently than late-required words [28]. The number of switches and the number of clusters are correlated, as by identifying the number of clusters, one can generate the number of switches (corresponds to the number of clusters minus one) [27].

Irrespective of the underlying FTD mutation, we showed a decline in the total score in mutation carriers from at least 4 years prior to phenoconversion. Decline in semantic fluency was found to be an early cognitive marker in other neurodegenerative diseases. For instance, in preclinical AD and Huntington’s disease, as early as 12 years before the onset of dementia, decline in a measure of semantic memory was found [29, 30]. In another study, it was amongst the most statistically sensitive cognitive measures of symptomatic conversion [31]. Studies into semantic fluency decline in presymptomatic FTD have shown somewhat contrasting results. One study demonstrated decline in semantic fluency in *MAPT* mutation carriers from 6 years before estimated symptom onset [7], whereas another only found decline at estimated symptom onset [6]. It should be noted that both studies used estimated years to symptom onset as a proxy for actual onset, which can be less reliable in familial FTD [6]. Utilizing a similar research design as our current study, decline of semantic fluency from 4 years before symptom onset in *MAPT* converters, and decline of semantic fluency was found to be the best predictor for having an *MAPT* mutation [8]. Although semantic fluency did not decline in *GRN* converters, decline on phonemic fluency was found to be predictive of an *GRN* mutation, confirming the value of fluency tasks in presymptomatic FTD as they can distinguish the underlying genotype [8].

Starting at least 4 years pre-phenoconversion, *GRN* mutation carriers produced fewer but larger clusters, and had fewer switches, than *MAPT* phenoconverters and controls. A likely explanation for the decline in their total score is that *GRN* phenoconverters deteriorate in cognitive flexibility [32], and thereby lose the ability to switch between semantic clusters in order to generate more words. Indeed, in our study the decline in the number of clusters and switches, and the increase in cluster size, correlated with decline in executive function. Executive dysfunction is known to be a distinctive cognitive feature in *GRN* mutations, demonstrating deficits in symptomatic mutation carriers [33], extending to the presymptomatic stage [7, 8]. When converting to the symptomatic stage, *GRN* mutation carriers also show the most decline in executive function [34].

Starting at least 4 years pre-phenoconversion, *MAPT* mutation carriers produced words with a higher LF and a lower AoA, and had fewer clusters and smaller clusters than *GRN* phenoconverters and controls. These findings point towards deterioration of the semantic system as an explanation as to why qualitative fluency measures change in *MAPT*. First, semantic decline is most likely to affect words with a lower LF and a higher AoA first, as the categorical organization of the system retrieves the ‘typical’ exemplars faster and more accurately, and they are better represented and more interconnected to other concepts than those that enter the semantic system later in life [35]. The reliance of both processes on semantic processing is further supported by their correlation with the verbal SAT which assesses verbal semantic deficits [36]. Clustering relies on lexical retrieval, vocabulary size and lexical access, and thus is mainly supported by the integrity of the semantic system [13].

We demonstrated that—irrespective of the underlying mutation—decline in the number of clusters, cluster size, and switches correlated with GM volume loss of predominantly frontal areas, while worse LF and AoA performance was associated with GM volume loss of predominantly temporal areas. These neuroanatomical correlates are in line with the predominant frontal involvement in *GRN* mutation carriers [6, 34], and link the degradation of the fronto-insula network to less cognitive flexibility—and as a consequence early clustering-switching impairment—as the most likely underpinning of declining fluency performance in conversion to *GRN*-associated FTD. The finding that LF and AoA rely on temporal lobe functioning could explain why these qualitative features are changing early in *MAPT* mutation carriers, as temporal volume loss is considered the neuroimaging hallmark of *MAPT* [37, 38], being present up to several decades before symptom onset [39]. Although PPA is not a frequent clinical phenotype, semantic impairments are well-described in *MAPT*-related FTD [40]. Our cohort includes three *P301L* and three *G272V* phenoconverters, which is too small to investigate differences between the two tau mutations. Nevertheless, with larger sample sizes it would be interesting to explore if there is clinical heterogeneity across the *MAPT* mutations [41], as the *P301L* mutation often presents with a language phenotype with semantic deficits [42]. Impairments in semantic fluency are found to be common as the result of cerebellar pathology, as this subcortical region plays a crucial role in motor performance and executive processes necessary for organizing and monitoring word output [43].

The key strength of our study is our longitudinal design, spanning up to 6 years of follow-up in a large single-centre sample of participants from *GRN* and *MAPT* FTD-families. This design allowed the investigation of mutation carriers as they were converting to the symptomatic stage, which provides us more accurate information about the underlying disease process than previous studies that used estimated years to onset as a proxy [40]. We chose multilevel linear modelling to handle potential missing data and unbalanced time-points that were the result of our ongoing prospective study. The small sample of phenoconverters, in combination with the large fluctuations in the data of the qualitative measures, are the largest drawback of the study, which has hampered our statistical power and interpretation of results. As the multilevel model assumes a linear relationship between genetic status and fluency performance it is possible that we have missed non-linear effects. Ideally, the semantic fluency test should not have been used in determining phenoconversion, however in our multidisciplinary approach we have used all available clinical information—e.g., MR brain imaging, anamnestic and heteroanamnestic information, questionnaires—so that symptom onset did not solely depend on the neuropsychological assessment. Although theoretically a cluster can consist of a single word, one cannot measure interword intervals for fewer than two words, so that a single-word “cluster” cannot be corroborated by the measurement of interword intervals. Following Ledoux et al. [11], we therefore defined clusters not as single words, but only as multiword strings (i.e. two or more consecutive words) whose relationship is defined by one of the scoring rules, but realize this could have penalized patients with a low total output. Lastly, the analyses on the presymptomatic mutation carriers were performed using the original baseline and follow-up visits, regardless of years from potential phenoconversion, therefore they might have lost some sensitivity to detect decline. Future directions include replication of our findings in larger multicentre cohorts, including *C9orf72* mutation carriers. Moreover, using qualitative measures in discriminative event-based models could help us understand the dynamics of disease progression and how other biomarkers (e.g., NfL) fit into this [44]. Lastly, future studies could look into the effect of using time-bins next to the usual 60-s output, as most people start with readily available animals and produce less familiar exemplars as the task develops (which affects the qualitative measures), and was found to be particularly sensitive to mutation status in presymptomatic *APOE*-ε4 carriers at-risk of developing AD [14].

### Conclusion

Our pilot study shows that qualitative aspects of semantic fluency change in presymptomatic FTD, and shows different profiles and inflection points depending on the mutation involved. This could provide important insight into the mechanisms as to why the “traditional” total score is declining. Its brief and easy-to-apply nature makes the total score of the semantic fluency test a likely candidate cognitive biomarker for upcoming clinical trials for FTD, but more research with a larger sample of phenoconverters is needed to replicate our findings, and to explore the additional value of qualitative measures in identifying and tracking mutation carriers as they convert to the symptomatic stage.

## Data Availability

Anonymized data not published within this article will be made available upon reasonable request from any qualified investigator.

## Appendix 1: Scoring guidelines for qualitative aspects of semantic fluency

### General scoring rules

When more than three words were missing from the fluency output (e.g., word was illegible or there was a tick instead of a word written), only the total number of words generated was used for further analyses (i.e., no qualitative aspects were calculated). For the total number of words generated, only correct words were used, thus no perseverative, pseudo or rule-break errors were included. Clusters were allowed to (partially) overlap. ‘Embedded’ clusters (i.e., a cluster within a cluster) were not taken into account. In determining the total cluster size, words that belonged to more than one cluster were counted twice (e.g., pig belongs to the cluster “farm animals” as well as the cluster “porcine”). In determining the number of switches, overlapping clusters were also counted as a switch. The beginning and end of the output were not taken into account as a switch.

### Ad hoc scoring rules for LF and AoA

For LF and AoA the original fluency output was followed if possible. If a word was not found in the databases for either LF [25] or AoA [26] the following *ad hoc *scoring rules were followed: 1. finding the singular of this word in the database (e.g., pig instead of pigs); 2. finding the word in the database with the adjective removed (e.g., bear instead of polar bear). When the word was still not available, a different value was used: for LF it was assumed that the missing word occurred only once in the language corpus. With this assumed frequency a Zipf-value of ‘’zero frequency’’ was calculated. For AoA, an imputed value was used when the word was not available in the database. The imputed value was based on the highest age of acquisition score (15.1) within this study.

### Cluster scoring rules*

**Table:**

Words that belong to the same subcategory	E.g., pets; dog, cat, rabbit
Words that have a strong association in the real world	E.g., cat, mouse
Words that share the same first letter	E.g., fish, ferret
Words that share the same first sound	E.g., cat, kangaroo
Words that share the same word	E.g., horse, seahorse
Words that rhyme	E.g. cat, rat

Based on Ledoux et al. [11] and Troyer et al. [24]

## Appendix 2: Principal component analysis on the five qualitative fluency measures

**Table:**

Items	Component coefficients		Communalities
	1	2	
4 years before phenoconversion			
LF	*− 0.958*	0.025	0.915
AoA	*0.950*	0.033	0.904
Clusters	0.061	*0.888*	0.792
Cluster size	0.535	*− 0.593*	0.637
Switching	− 0.014	*0.907*	0.824
Eigenvalues	2.314	1.758	
Variance, %	46.280	35.167	
2 years before phenoconversion			
LF	*0.975*	0.025	0.951
AoA	*0.970*	− 0.024	0.941
Clusters	0.172	*0.837*	0.731
Cluster size	0.235	*− 0.807*	0.706
Switching	0.039	*0.902*	0.815
Eigenvalues	1.977	2.167	
Variance, %	39.542	43.332	
Phenoconversion			
LF	*− 0.942*	0.034	0.888
AoA	*0.948*	− 0.137	0.918
Clusters	0.424	0.093	0.188
Cluster size	0.228	*− 0.839*	0.756
Switching	0.249	*0.855*	0.793
Eigenvalues	2.096	1.448	
Variance, %	41.922	28.965	
1 year after phenoconversion			
LF	*− 0.959*	− 0.015	0.920
AoA	*0.950*	0.103	0.913
Clusters	0.047	− 0.350	0.629
Cluster size	*0.711*	*0.919*	0.847
Switching	− 0.124	*0.924*	0.869
Eigenvalues	2.420	1.758	
Variance, %	48.396	35.156	
2 years after phenoconversion			
LF	*− 0.929*	− 0.220	0.912
AoA	*0.938*	0.168	0.907
Clusters	0.197	*0.903*	0.853
Cluster size	*0.606*	*− 0.624*	0.756
Switching	0.196	*0.904*	0.855
Eigenvalues	2.484	1.800	
Variance, %	49.679	35.995	

Abbreviations: *LF* lexical frequency, *AoA* Age of Acquisition

## Appendix 3: Neuroimaging correlates of the qualitative fluency measures

**Table:**

Group	Measure	Cluster	*T*	*P*_FWE-corrected_	MNI coordinates			Region
					*x*	*y*	*z*	
Total group of phenoconverters	LF	71,256	73.01	< 0.001	33	20	− 4	Anterior insula R
		851	77.34	< 0.001	− 33	16	52	Middle frontal gyrus L
		639	49.57	< 0.001	− 20	42	34	Superior frontal gyrus L
		476	81.60	< 0.001	− 33	16	54	Middle frontal gyrus L
		438	32.65	< 0.001	56	− 45	2	Middle temporal gyrus R
		426	26.97	< 0.001	60	− 26	0	Superior temporal gyrus R
		124	38.36	< 0.001	− 24	2	− 48	Temporal pole L
		117	27.64	0.002	− 22	− 3	− 46	Inferior temporal gyrus L
	AoA	98,828	88.54	< 0.001	− 33	16	56	Middle frontal gyrus L
		1038	37.85	< 0.001	− 32	42	30	Middle frontal gyrus L
		442	32.71	< 0.001	57	− 42	4	Middle temporal gyrus R
		425	28.39	< 0.001	60	− 26	0	Superior temporal gyrus R
		348	24.14	< 0.001	54	− 60	− 8	Inferior temporal gyrus R
		171	31.55	< 0.001	− 32	56	12	Middle frontal gyrus L
	Clusters	10,400	70.87	< 0.001	− 33	− 6	9	Anterior insula L
		774	66.01	< 0.001	63	− 38	30	Supramarginal gyrus R
		401	58.95	< 0.001	− 34	18	57	Middle frontal gyrus L
		341	24.59	0.005	54	− 60	− 8	Inferior temporal gyrus R
		254	40.05	< 0.001	− 32	42	30	Middle frontal gyrus L
		191	35.09	< 0.001	− 51	− 48	18	Superior temporal gyrus L
		135	24.82	0.005	− 4	32	48	Superior frontal gyrus L
	Cluster size	80,154	89.77	< 0.001	− 3	9	38	Middle cingulate gyrus L
		1513	37.39	< 0.002	56	− 45	3	Middle temporal gyrus
		714	44.30	< 0.001	63	− 38	30	Supramarginal gyrus R
		390	24.13	< 0.001	57	− 63	− 2	Inferior temporal gyrus R
		265	39.38	0.002	− 32	42	30	Middle frontal gyrus L
		232	27.25	0.006	− 21	39	40	Superior frontal gyrus L
	Switches	70,083	67.98	< 0.001	33	18	− 4	Anterior insula R
		1420	31.22	0.001	8	16	57	Supplementary motor cortex R
		766	44.18	< 0.001	63	− 38	28	Supramarginal gyrus R
		502	33.31	< 0.001	57	− 42	4	Middle temporal gyrus R
		487	58.10	< 0.001	− 34	18	57	Middle frontal gyrus L
		435	28.07	0.002	46	− 52	8	Inferior temporal gyrus R
		373	26.80	0.003	62	− 18	− 3	Superior temporal gyrus R
		286	50.52	< 0.001	44	− 51	48	Angular gyrus R
		277	29.04	0.001	− 50	− 63	− 32	Cerebellum L
		276	60.77	< 0.001	− 33	42	32	Middle frontal gyrus L
		179	28.99	0.001	− 22	39	42	Superior frontal gyrus L
*GRN* phenoconverters	LF	122	114.52	0.002	3	− 52	− 12	Cerebellum R
		72	99.72	0.002	− 30	0	− 18	Medial temporal lobe L
		60	114.68	0.002	27	− 44	− 24	Cerebellum R
		32	92.53	0.002	40	− 57	− 42	Cerebellum R
		28	111.21	0.002	− 20	− 46	− 21	Cerebellum L
		25	93.84	0.002	− 34	10	− 10	Anterior insula L
		18	95.61	0.002	− 39	− 78	− 33	Cerebellum L
	AoA	142	113.75	0.002	0	− 52	− 14	Cerebellum L
		85	116.16	0.002	24	− 44	− 24	Cerebellum R
		55	96.63	0.002	− 38	− 8	6	Anterior insula L
		38	91.25	0.002	− 28	0	− 16	Medial temporal lobe L
		23	106.62	0.002	− 20	− 46	− 22	Cerebellum L
		15	93.90	0.002	− 57	− 46	− 21	Inferior temporal gyrus L
	Clusters	328	133.16	0.002	18	− 66	− 21	Cerebellum R
		141	127.04	0.002	− 38	− 9	− 4	Posterior insula L
		119	122.36	0.002	− 2	− 52	− 10	Cerebellum L
		106	128.52	0.002	− 20	− 52	− 20	Cerebellum L
		64	109.98	0.002	− 20	8	− 12	Putamen L
		20	106.12	0.002	21	− 38	− 26	Cerebellum R
		17	113.55	0.002	33	20	− 4	Anterior insula R
	Cluster size	150	119.60	0.002	0	− 52	− 14	Cerebellum L
		89	111.33	0.002	− 36	− 9	− 4	Posterior insula L
		72	117.38	0.002	24	− 44	− 22	Cerebellum R
		43	102.41	0.002	− 20	8	− 12	Putamen L
		11	98.60	0.002	33	21	− 4	Anterior insula R
	Switches	114	112.68	0.002	− 3	− 52	− 14	Cerebellum L
		104	113.17	0.002	− 22	− 50	− 24	Cerebellum L
		69	121.55	0.002	− 22	9	− 6	Putamen L
		57	115.68	0.002	− 36	− 4	− 6	Posterior insula L
		35	103.79	0.002	5	− 50	− 16	Cerebellum R
		33	104.91	0.002	21	− 39	− 24	Cerebellum R
		27	102.79	0.002	− 24	− 74	− 21	Cerebellum L
*MAPT* phenoconverters	LF	25,864	143.78	< 0.001	22	− 84	− 27	Cerebellum R
		117	18.41	< 0.001	33	22	− 40	Inferior temporal gyrus R
		58	23.94	< 0.001	40	− 4	− 51	Temporal pole R
		38	29.20	< 0.001	− 32	20	− 30	Temporal pole L
		35	16.10	< 0.001	− 39	21	− 38	Temporal pole L
		28	16.07	0.002	51	21	− 21	Temporal pole R
	AoA	26,431	147.06	< 0.001	22	− 84	− 27	Cerebellum R
		119	18.43	< 0.001	33	22	− 40	Temporal pole R
		47	23.08	< 0.001	39	− 6	− 51	Inferior temporal gyrus R
		27	15.77	< 0.001	51	21	− 20	Temporal pole R
	Clusters	22,010	144.32	< 0.001	22	− 84	− 28	Cerebellum R
		1669	68.58	< 0.001	− 32	44	30	Middle frontal gyrus L
		342	57.46	< 0.001	45	24	42	Middle frontal gyrus R
		310	24.65	< 0.001	4	58	32	Superior frontal gyrus R
		224	33.39	< 0.001	− 4	60	18	Superior frontal gyrus L
		216	56.60	< 0.001	38	46	28	Middle frontal gyrus R
		201	25.60	< 0.001	51	42	14	Middle frontal gyrus R
		134	24.15	< 0.001	36	28	54	Middle frontal gyrus R
	Cluster size	21,760	146.75	< 0.001	34	− 42	− 30	Cerebellum R
		1509	71.70	< 0.001	− 32	44	28	Middle frontal gyrus L
		382	43.23	< 0.001	45	24	44	Middle frontal gyrus R
		238	28.06	< 0.001	− 4	60	20	Superior frontal gyrus L
		236	21.36	< 0.001	20	63	− 16	Frontal pole R
		228	24.25	< 0.001	4	58	32	Superior frontal gyrus R
		212	43.14	< 0.001	− 21	44	42	Superior frontal gyrus L
		194	23.61	< 0.001	50	40	14	Middle frontal gyrus R
		185	25.34	< 0.001	− 46	52	− 2	Middle frontal gyrus L
		167	44.46	< 0.001	38	48	28	Middle frontal gyrus R
		103	20.98	< 0.001	36	28	52	Middle frontal gyrus R
	Switches	22,641	152.76	< 0.001	24	− 82	− 28	Cerebellum R
		297	37.51	< 0.001	44	26	42	Middle frontal gyrus R
		173	38.47	< 0.001	26	51	36	Superior frontal gyrus R
		149	25.54	< 0.001	4	58	32	Superior frontal gyrus R
		144	20.55	< 0.001	34	21	− 39	Temporal pole R
		132	24.69	< 0.001	− 21	42	45	Superior frontal gyrus L

Abbreviations: *LF* lexical frequency, *AoA* Age of Acquisition, *GRN* progranulin, *MAPT* microtubule-associated protein tau, *L* left, *R* right

## Footnotes

1 We used the scoring rules of Ledoux et al. [11] here, in which the scoring of clusters is restricted to strings of two or more successive words. Although theoretically a cluster can consist of only a single word, one cannot measure interword intervals for fewer than two words, thus a single-word cluster cannot be corroborated by the measurement of interword intervals. Reference: Ledoux et al. [11].
